# Application and comparison of different implanted ports in malignant tumor patients

**DOI:** 10.1186/s12957-016-1002-6

**Published:** 2016-09-23

**Authors:** Yanhong Li, Yonghua Cai, Xiaoqin Gan, Xinmei Ye, Jiayu Ling, Liang Kang, Junwen Ye, Xingwei Zhang, Jianwei Zhang, Yue Cai, Huabin Hu, Meijin Huang, Yanhong Deng

**Affiliations:** Department of Medical Oncology, The Sixth Affiliated Hospital, Sun Yat-sen University, Guangzhou, Guangdong 510655 China

**Keywords:** Vein port, Complication, Upper arm vein, Jugular vein, Subclavian vein

## Abstract

**Background:**

The current study aims to compare the application and convenience of the upper arm port with the other two methods of implanted ports in the jugular vein and the subclavian vein in patients with gastrointestinal cancers.

**Methods:**

Currently, the standard of practice is placement of central venous access via an internal jugular vein approach. Perioperative time, postoperative complications, and postoperative comfort level in patients receiving an implanted venous port in the upper arm were retrospectively compared to those in the jugular vein and the subclavian vein from April 2013 to November 2014.

**Results:**

Three hundred thirty-four patients are recruited for this analysis, consisting of 107 in the upper arm vein group, 70 in the jugular vein group, and 167 in the subclavian vein group. The occurrence of catheter misplacement in the upper arm vein is higher than that in the other two groups (13.1 vs. 2.9 vs. 5.4 %, respectively, *P* = 0.02), while the other complications in the perioperative period were not significantly different. The occurrence of transfusion obstacle of the upper arm vein group is significantly lower than that of the jugular and subclavian groups (0.9 vs. 7.1 vs. 7.2 %, *P* = 0.01). The occurrence of thrombus is also lower than that of other two groups (0.9 vs. 4.3 vs. 3.6 %, *P* = 0.03). Regarding the postoperative comfort, the influences of appearance (0 vs. 7.1 vs. 2.9 %, *P* = 0.006) and sleep (0.9 vs. 4.2 vs. 10.7 %, *P* = 0.003) are significantly better than those of the jugular and subclavian vein groups.

**Conclusions:**

Compared to the jugular and the subclavian vein groups, the implanted venous port in the upper arm vein has fewer complications and more convenience and comfort, and might be a superior novel choice for patients requiring long-term chemotherapy or parenteral nutrition.

## Background

The incidences of gastrointestinal cancers are increasing. Most of the patients require long-term chemotherapy or have to receive 5-fluorouracil-based chemotherapy. Therefore, more and more patients need a central venous catheter in order to protect the peripheral vein. Totally implantable central venous ports (CVP) comprise all the devices embedded in the subcutaneous tissue, protected by skin. CVP with the minimum limit to daily life applies to patients requiring long-term repeated venipuncture, chemotherapy, parenteral nutrition support, and transfusion [1].

The jugular vein and the subclavian vein are commonly selected with the injection base embedded under the clavicle and chest skin. However, these two methods carry a risk of puncture resulting to pneumothorax, hemothorax, and artery injury [2, 3]. Currently, the standard of practice is placement of central venous access via an internal jugular vein approach. The arm port is a novel implanted port method, which only requires selecting the basilica vein or brachial vein, guided by vessel B ultrasonic set, for puncture by improved Seldinger puncture technique in the peripherally inserted central catheter (PICC) room. This method solely requires a professional nurse for vein therapy with PICC catheterizing qualification for operation under the doctor’s supervision. The whole procedure is simple and avoids occupancy of the operating room. Any large cohort of patients undergoing the new method has not been widely reported. The current study aims to compare the operation time, occurrence of postoperative complications, and patient comfort of the upper arm port with the other two methods of implanted ports in the jugular vein and the subclavian vein in patients with gastrointestinal cancers.

## Methods

### Patients

A retrospective analysis was conducted for patients receiving implantation of three different access ports in The Sixth Affiliated Hospital of Sun Yat-sen University, Medical Oncology, from April 2013 to November 2014. All of these patients were pathologically diagnosed with gastrointestinal cancer. Follow-up starts from the day of port implantation until the day of removal for any reasons or patients’ death. Admission criteria are as follows: (1) all the patients should be more than 18 years, with malignant tumor confirmed pathologically; (2) examination of the patients heart, liver, kidney, and blood should be normal; (3) patients should have chemotherapeutic indications without chemotherapeutic and venous catheterization contraindications; and (4) the arm circumference for implantation port on the arm should be ≥25 cm. Exclusion criteria are as follows: (1) any patient who has been confirmed or suspected of infection, bacteremia, or blood poisoning; (2) patients with too small body size to accept implantation equipment; (3) patients with chronic obstructive pulmonary disease; and (4) patients with radiotherapy, trauma, and surgery at the operative site. This study was approved by the institutional review board of The Sixth Affiliated Hospital, Sun Yat-sen University (Guangzhou, Guangdong), and written informed consent was obtained from every participant.

### Device for access port

The device for access port is 6F fine implanted port suite manufactured by US Bard Co., Ltd. with a catheter length of 75 cm, and the device for the jugular venous and subclavian venous ports is 7.5F implanted access port suite manufactured by Braun Medical Co., Ltd. with a catheter length of 50 cm.

### Implantation of access port

Implantation of access port should be conducted with local anesthesia. Access port on the arm should be implanted by two professional nurses with PICC implantation qualification. Basilica or cephalic vein of the upper right arm is selected for puncture guided by the color vessel Doppler ultrasound with improved Seldinger puncture technique. Subsequent to catheter implantation, routine chest X-ray should be examined for the feeding of the catheter and the position of the tip. If the catheter reverses or the tip position is abnormal, it should be readjusted with the aid of X-rays. Under a physician’s guidance, the nurses transect the skin about 3 cm with the center of the puncture point to split the subcutaneous tissue for the capsular bag and embed the port foundation under the skin of the upper arm without establishing the subcutaneous tunnel.

Jugular venous access and subclavian venous access ports should be implanted by qualified physicians into the right jugular vein or subclavian vein with puncture point 2 cm below the middle point between the right cleidomastoid sternal branch and top or right clavicle at the clavicle angle. After implanting the catheter, a subcutaneous tunnel is established on the right chest wall. Subsequently, a routine chest X-ray film is examined for the feeding and the catheter position.

### Maintenance and follow-up of catheter

Fluid can be injected after implanting the access port. Intact butterfly needle is used to puncture into the port foundation after skin disinfection. Normal saline is used to wash the catheter to detect an obstacle and subcutaneous seepage in the catheter. When consecutive infusion is required, intact butterfly needle should be replaced every week. Upon completion of the infusion therapy, 10 ml heparin saline (100 IU/ml) is used to wash the catheter. If the catheter cannot be used for more than 4 weeks, it should be washed in the hospital every 4 weeks. Follow-up starts from the port implantation until the removal surgery, and the withdrawal date should be treated as the final usage date of the infusion port.

### Collection of complications

Major observation indices refer to the perioperative time of three different access ports (the date of port implantation) and incidence rates of postoperative complications by recording the types and the time of complication incidence. The complications in the perioperative period include catheter misplacement, pneumothorax, hemothorax, artery injury, and failed puncture, which requires a change of the puncture path. The postoperative complications include infection, thrombogenesis, poor transfusion, catheter breakage, pinch-off syndrome, seepage on foundation, and foundation exposure. The investigation of postoperative comfort is carried out in a subsequent visit (after 15 days); nurses who do not attend this study inquire and record the patient’s appearance effects, concerns about catheter breakage, and effect on sleep and daily life.

### Statistical method

Perioperative time, postoperative complications, and postoperative comfort level in patients receiving an implanted venous port in the upper arm were retrospectively compared to those in the jugular vein and the subclavian vein. The statistical software is SPSS19.0. Statistical data is represented as a mean ± standard deviation. ANOVA is used for mean comparison, and LSD of post hoc inspection is used for the comparison of two averages. Chi-squared test is used to compare the complication incidence. *P* < 0.05 is considered statistically significant.

## Results

### General condition of the patients

The analysis recruited 344 patients, including 107 patients in the upper arm vein group, 70 in the jugular vein group, and 167 in the subclavian vein group. Table 1 shows the clinical characteristics of patients in the three groups, with similar basic information. In the three groups, the average follow-up durations were 257.6 ± 134.1, 307.9 ± 134.3, and 253.4 ± 152.8 days (*p* = 0.03), and seven, two, and nine patients failed the follow-up, respectively.

**Table 1:** Baseline characteristics

Variable	Upper vein group	Jugular vein group	Subclavian vein group
Patients	107	70	167
Age (years)	54.4 ± 12.3	53.8 ± 13.0	53.0 ± 13.3
Sex			
Male	64 (59.8)	41 (58.6)	104 (62.3)
Female	43 (40.2)	29 (41.4)	63 (37.7)
Primary tumor			
Colon cancer	33 (30.8)	31 (44.3)	66 (39.5)
Rectal cancer	52 (48.6)	31 (44.3)	84 (50.3)
Gastric cancer	12 (11.2)	7 (10.0)	13 (7.8)
Pancreatic cancer	2 (1.9)	1 (1.4)	0 (0)
Small intestinal carcinoma	1 (0.9)	0 (0)	0 (0)
Esophageal cancer	1 (0.9)	0 (0)	3 (1.8)
Bile duct carcinoma	1 (0.9)	0 (0)	0 (0)
Hepatic carcinoma	1 (0.9)	0 (0)	0 (0)
Others	4 (3.7)	0 (0)	1 (0.6)
Follow-up duration (days)	257.6 ± 134.1	307.9 ± 134.3	253.4 ± 152.8

Data in parenthesis is a percentage

### Complications in ports in the perioperative period

The patients in all the groups have successfully received port implantation. Patients in the three groups prefer right implantation. Complications in the operation include catheter misplacement, pneumothorax, hemothorax, artery injury, and failed puncture or catheterizing, which requires changing puncture access. The upper vein group should take the left upper vein access, while the jugular and subclavian vein groups should take the subclavian access and jugular access, respectively, because of failed puncture or catheterizing. The incidence of catheter misplacement in the upper arm vein group is higher than that of the other two groups (13.1 vs. 2.9 vs. 5.4 %, *P* = 0.02), while the other complications in the perioperative period do not alter significantly, as shown in Table 2.

**Table 2:** Complications in the perioperative period

Complications	Upper arm vein group	Jugular vein group	Subclavian vein group	*P* value
Catheter misplacement	14 (13.1)	2 (2.9)	9 (5.4)	0.02
Pneumothorax	0 (0)	1 (1.4)	2 (1.2)	0.50
Hemothorax	0	0	0	
Artery injury	1 (0.9)	4 (5.9)	6 (3.6)	0.18
Change of puncture access	10 (9.3)	3 (4.3)	9 (5.4)	0.31

Data in parenthesis is a percentage

### Postoperative complications

Table 3 shows complications after port implantation. Any differences in the complications of foundation exposure, seepage in the foundation, infection, and thrombus among the three groups were not observed. However, the incidence of poor transfusion in the upper vein group is significantly lower than that in the jugular and subclavian vein groups (0.9 vs. 7.1 vs. 7.2 %, *P* = 0.01), and its incidence of thrombus was also lower (0.9 vs. 4.3 vs. 3.6 %, *P* = 0.03); 3, 5, and 13 patients required the removal of the port because of complications, without any statistical significance (*P* = 0.23).

**Table 3:** Complications after port implantation

Complications	Upper arm vein group	Jugular vein group	Subclavian vein group	*P* value
Foundation exposure	2 (1.9)	1 (1.4)	0 (0)	0.23
Seepage on foundation	1 (0.9)	1 (1.4)	1 (0.6)	0.82
Thrombus	1 (0.9)	3 (4.3)	6 (3.6)	0.03
Infection	1 (0.9)	2 (2.9)	6 (3.6)	0.40
Poor transfusion	1 (0.9)	5 (7.1)	12 (7.2)	0.01
Catheter breakage	0 (0)	0 (0)	2 (1.2)	0.34
Pinch-off syndrome	0 (0)	0 (0)	3 (1.8)	0.20
Patients with pump removal	3 (2.8)	5 (7.1)	13 (7.8)	0.23

Data in parenthesis is a percentage

### Postoperative comfort after port implantation

The comparison of postoperative comfort shows that the appearance concern (0 vs. 7.1 vs. 2.9 %, *P* = 0.006) and the sleep influence (0.9 vs. 4.2 vs. 10.7 %, *P* = 0.003) in the upper arm vein group were not significant as compared to those in the jugular and subclavian vein groups (Table 4).

**Table 4:** Comparison of postoperative comfort after port implantation

Items	Upper arm vein group	Jugular vein group	Subclavian vein group	*P* value
Appearance	0 (0)	5 (7.1)	5 (2.9)	0.006
Concern of catheter breakage	3 (2.8)	4 (5.7)	18 (10.7)	0.19
Sleep influence	1 (0.9)	3 (4.2)	18 (10.7)	0.003
Influence on daily life	0 (0)	0 (0)	2 (1.1)	0.26

Data in parenthesis is percentage

## Discussion

The current study compared the application of the upper arm venous port, the jugular venous port, and the subclavian venous port in patients with malignant tumor. The result shows that there is no significant difference in the perioperative period among the three groups. While the incidence of the postoperative complications in the upper arm venous group is lower, the comfort level is higher.

Implantation of the upper arm port is conducted in the PICC catheterizing room; hence, the catheter feeding and tip position cannot be determined during the operation. Moreover, because the catheterizing route is elongated, it is easy to mislead the feeding to the jugular vein, thus reversing the catheter, or catheterizing deeply into the heart to cause discomfort in the neck or precordial area. Therefore, we measure the distance between the planned puncture point and the sternum midpoint in a preoperative routine as the reference depth of catheterization during operation. Meanwhile, routine inspection with a chest X-ray film is conducted to distinguish the catheter feeding and tip position after catheterizing, rather than cutting the skin to implant the foundation. If the catheter position is abnormal, the adjustment should be conducted under the X-ray machine. Although the incidence of catheter misplacement is high (13.1 %) in the upper arm vein group, the position can be adjusted to normal under perspective. Also, without a subcutaneous tunnel, the complication of poor transfusion is also lower than that in the jugular and subclavian groups (*P* = 0.06). The lumen of the upper arm vein is remarkably small, such that the puncture failure rate was reported to be 5–11 % [4, 5], and the ratio of changing puncture access in this group was 9.3 %. Kawamura et al. [6] assessed the implantation of the upper arm port in 113 patients with metastatic colorectal cancer receiving chemotherapy. All the patients received puncture under the guidance of real-time ultrasound or radiation, and none of them demonstrated complications in the perioperative period; only nine patients showed postoperative complications (8.0 %). It was concluded that the upper arm port exhibited favorable short- and long-term effects.

The upper arm port access can completely avoid severe puncture complications, such as pneumothorax and hemothorax, and only one patient had artery injury (0.9 %). The blind puncture was conducted in the jugular and subclavian vein groups, which is greatly influenced by the experience of operators and individual anatomic differences. Thus, the probability of pneumothorax (0.3–3.2 %) [7, 8] and artery injury is high. The incidence of catheter misplacement is low when choosing the jugular vein as the puncture access, but is simple to distort and narrow for the catheter to cause poor transfusion. Since the injection foundation is embedded on the upper wall, the catheter should be downward feeding after turning 180° and the subcutaneous tunnel elongated. Moreover, the tractive feeling in the neck after the operation is obvious, lowering the comfort level. The subcutaneous tunnel in the subclavian venous access is short. Thus, the patients have no tractive feeling in the neck. However, severe complications, such as pneumothorax, hemothorax, mediastinum hematoma, and pinch-off syndrome may occur in the puncture to cause catheter breakage [9, 10]. When the catheter passes through the interval between the clavicle and the first rib, poor transfusion, thrombus, breakage, and perforation may occur, and the catheter may even rupture because of pressure. The broken end may flow into the heart or lung along with the blood flow to cause arrhythmia or pulmonary embolism. In this study, three patients had a pinch-off syndrome in the subclavian vein group (1.8 %), including two patients requiring emergency surgery for the removal of the broken end due to catheter fracture.

In the postoperative complications, the incidence of infection in the upper arm vein group (0.9 %) is lower than that in the jugular vein group and subclavian vein group. The study did not record any significant difference in the infection rates of arm port and subclavian venous port [11, 12]. In the long-term maintenance of ports, phlebothrombosis is another severe complication. Patients and port implantation may increase the risk of phlebothrombosis [13]. The retrospective study shows that the incidence of thromboembolism related to the catheter is up to 12–64 %, but the anticoagulant for routine prevention is not recommended [14], even though one study indicates that the incidence of deep venous thrombosis in the arm port is higher than that in the chest port [15].

The other significant advantages of the upper arm port are that the postoperative comfort level and life quality of patients are high. The foundation embedded in the upper arm is concealed, and the invisibility of the puncture point and operation scar in the neck and chest renders it esthetically applicable for patients.

Moreover, in fluid replacement therapy, patients do not require upper body undressing, which avoids embarrassment and discomfort, especially in women [6]. Goltz et al. [16] found that the satisfaction and life quality of patients receiving arm port implantation were significantly higher than those of chest port implantation. Compared with the forearm port, the upper arm port has no difference in the infection rate, deep venous thrombosis, catheter misplacement, or stoppage. The embedded position of an upper arm port is high; short sleeves can cover the injection foundation without the subcutaneous tunnel, through chelidon. Therefore, we think the patients’ quality of life with the upper arm port is better.

The limitation of this study is that the type assessment of perioperative period and postoperative complications in the retrospective analysis is not quite comprehensive. Moreover, this is a study conducted by a single center, and patients in the three groups are not randomized. Also, the learning curve should be accomplished at an early stage after introducing the upper arm port into the hospital, to prevent the high incidences of catheter misplacement. However, few relevant studies on the upper arm are conducted in China, but several occur abroad. The current study provided evidence to assess the safety of clinical application of the upper arm port.

## Conclusions

In conclusion, the operation of the upper arm venous port is safe such that severe puncture complications, such as pneumothorax, hemothorax, and pinch-off syndrome, are avoided. The incidence of postoperative complications is lower than that in jugular venous access port and subclavian access port, and the discomfort level and patients’ quality of life is better. Therefore, the upper arm venous port implantation method might be a good choice in clinical applications.

## Abbreviations

CVP: Central venous ports
PICC: Peripherally inserted central catheter
